# Comparative molecular field analysis and molecular dynamics studies of α/β hydrolase domain containing 6 (ABHD6) inhibitors

**DOI:** 10.1007/s00894-015-2789-8

**Published:** 2015-09-08

**Authors:** Agnieszka A. Kaczor, Katarzyna M. Targowska-Duda, Jayendra Z. Patel, Tuomo Laitinen, Teija Parkkari, Yahaya Adams, Tapio J. Nevalainen, Antti Poso

**Affiliations:** Department of Synthesis and Chemical Technology of Pharmaceutical Substances with Molecular Modeling Laboratory, Faculty of Pharmacy with Division of Medical Analytics, Medical University of Lublin, 4a Chodźki St., 20059 Lublin, Poland; School of Pharmacy, University of Eastern Finland, Yliopistonranta 1, PO Box 1627, 70211 Kuopio, Finland; Department of Biopharmacy, Faculty of Pharmacy with Division of Medical Analytics, Medical University of Lublin, 4a Chodźki St., 20059 Lublin, Poland

**Keywords:** ABHD6, ABHD6 inhibitors, CoMFA, The endocannabinoid system, Homology modeling, Molecular docking, Molecular dynamics

## Abstract

The endocannabinoid system remains an attractive molecular target for pharmacological intervention due to its roles in the central nervous system in learning, thinking, emotional function, regulation of food intake or pain sensation, as well as in the peripheral nervous system, where it modulates the action of cardiovascular, immune, metabolic or reproductive function. α/β hydrolase domain containing 6 (ABHD6)—an enzyme forming part of the endocannabinoid system—is a newly discovered post-genomic protein acting as a 2-AG (2-arachidonoylglycerol) serine hydrolase. We have recently reported a series of 1,2,5-thiadiazole carbamates as potent and selective ABHD6 inhibitors. Here, we present comparative molecular field analysis (CoMFA) and molecular dynamics studies of these compounds. First, we performed a homology modeling study of ABHD6 based on the assumption that the catalytic triad of ABHD6 comprises Ser148–His306–Asp 278 and the oxyanion hole is formed by Met149 and Phe80. A total of 42 compounds was docked to the homology model using the Glide module from the Schrödinger suite of software and the selected docking poses were used for CoMFA alignment. A model with the following statistics was obtained: *R*^2^ = 0.98, *Q*^2^ = 0.55. In order to study the molecular interactions of the inhibitors with ABHD6 in detail, molecular dynamics was performed with the Desmond program. It was found that, during the simulations, the hydrogen bond between the inhibitor carbonyl group and the main chain of Phe80 is weakened, whereas a new hydrogen bond with the side chain of Ser148 is formed, facilitating the possible formation of a covalent bond.

**Electronic supplementary material:**

The online version of this article (doi:10.1007/s00894-015-2789-8) contains supplementary material, which is available to authorized users.

**Graphical Abstract:**
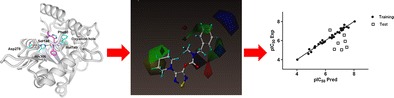
*Left*–*right*: Docking pose of **1** in the binding pocket of α/β hydrolase domain containing 6 (ABHD6) selected for molecular alignment; CoMFA steric and electrostatic contour fields; changes in potential energy of the complex during simulations for the complex of **6** and ABHD6

## Introduction

The endocannabinoid signaling system (ECS) regulates diverse physiological processes and has attracted significant attention as a potential drug target [1]. The ECS is engaged in many pathophysiological conditions in central and peripheral tissues. It is involved in the hormonal regulation of food intake, cardiovascular, gastrointestinal, immune, behavioral, antiproliferative and mammalian reproductive functions [2]. The ECS has also been linked with drug addiction [3] and alcoholism [4] while the dysregulation of ECS has been correlated to obesity and metabolic syndrome pathogenesis [5]. Also, the ECS has been linked to previously characterized phenomena called depolarization-induced suppression of inhibition (DSI) and depolarization-induced suppression of excitation (DSE) [6, 7]. Via DSI/DSE, the ECS acts as an important retrograde modulator system controlling the extent of neuronal excitability.

The key ligands of the endocannabinoid system are the lipid transmitters *N*-arachidonoylethanolamine (anandamide) and 2-arachidonoylglycerol (2-AG), which activate the two major cannabinoid receptors CB_1_ and CB_2_ [1, 2]. According to current knowledge, the lifetime of 2-AG is regulated by three enzymes belonging to the metabolic serine hydrolase family. Of these, monoacylglycerol lipase (MGL) is, on a quantitative basis, the main 2-AG hydrolase [8–10]. Two other hydrolases, α/β-hydrolase domain containing 6 (ABHD6) and 12 (ABHD12) have been identified recently [11, 12]. They are responsible for approximately 15 % of 2-AG hydrolysis in the brain. ABHD6 is an integral membrane protein of 30 kDa [13]. The active site of ABHD6 is suggested to be directed into the interior of the cell, which would make it possible to control the level of intracellular 2-AG [13]. Although the physiological and pathophysiological significance of this enzyme is largely unknown, it is proposed that high expression of ABHD6 is linked to some forms of cancer [13].

**Fig. 1:**
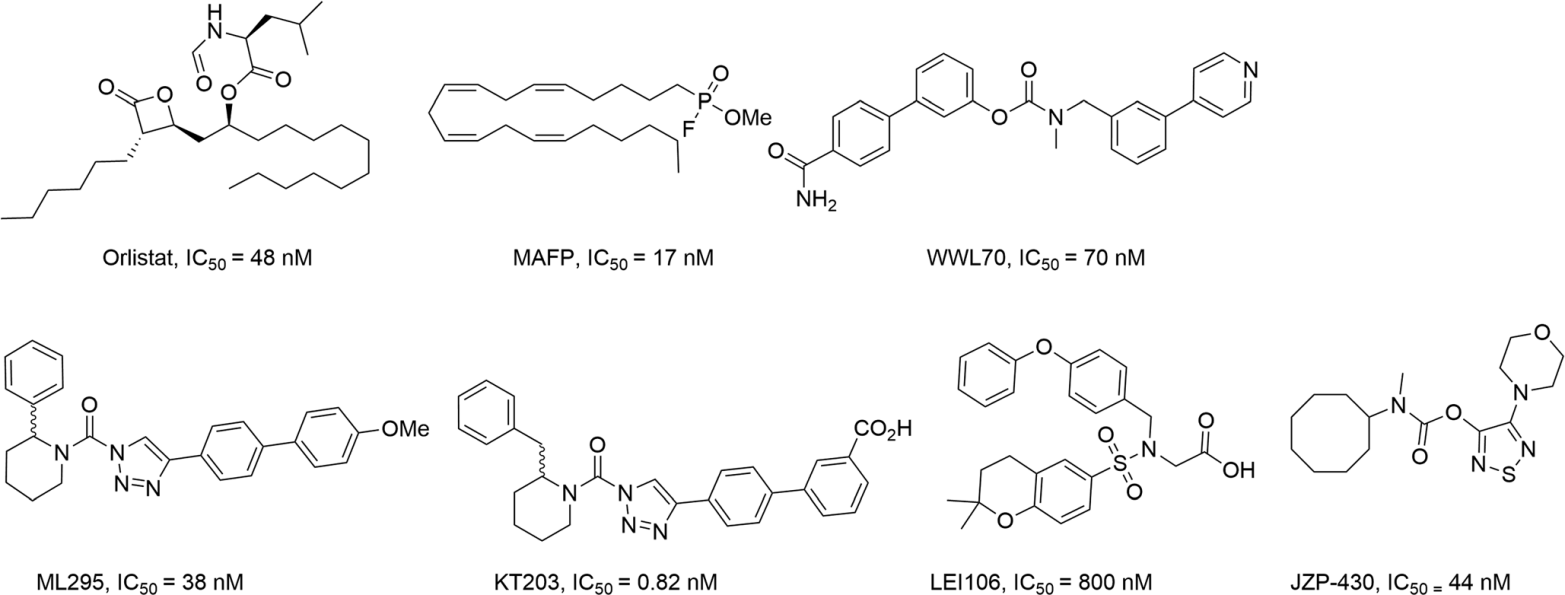
Known inhibitors of α/β-hydrolase domain containing 6 (ABHD6) [14–19]

Unlike MGL, ABHD6 and ABHD12 are still poorly characterized, due mainly to the lack of selective inhibitors. Their more detailed physiological and pathophysiological mapping requires development of highly selective and potent pharmacological tools. To date, only a few inhibitors of ABHD6 have been reported (Fig. 1) [14–19].

Modern computer-aided approaches can be helpful in the discovery of compounds with designed affinity profiles. Quantitative structure–activity relationships (QSAR) have been used extensively in the development of relationships between the physicochemical properties of chemical entities and their biological activities to arrive at reliable statistical models for the prediction of activities of novel chemical compounds [20]. The key assumption in this approach is that changes in structural properties are connected with differences in the biological activities of the compounds [20]. Three-dimensional (3D)-QSAR has been developed as a logical continuation of the widely used Hansch and Free-Wilson methods. It examines the 3D features of the compounds to predict their biological activities, applying robust chemometric techniques such as partial least squares (PLS), genetic partial least squares (G/PLS), artificial neural network (ANN), etc. [20]. 3D-QSAR approaches, including comparative molecular field analysis (CoMFA) [21] and comparative molecular similarity index analysis (CoMSIA) [22] make an important contribution to drug design through deriving structural information in interactive fields and predicting the influence on activity [23]. In particular, CoMFA is a well-established technique of 3D-QSAR [20, 24]. To the best of our knowledge, our study is the first attempt to develop a CoMFA model for ABHD6 inhibitors.

Nowadays, diverse techniques of molecular dynamics (MD) constitute important computational tools with which to study protein drug targets and their interactions with ligands at a molecular level, particularly for examining the motion of individual atoms, which can be tracked over time [23]. In spite of successful attempts to construct homology models of ABHD6 [19, 25], and to use molecular docking to build complexes with inhibitors, no MD studies have been reported for this enzyme.

We have recently developed a series of 1,2,5-thiadiazole carbamates as potent and selective ABHD6 inhibitors [19]. Here, we present CoMFA and MD studies of these compounds and several novel compounds.

## Computational methods

### Homology modeling and molecular docking

A homology model of ABHD6 was constructed as previously reported using Discovery Studio v. 3.5 [19]. We assumed in our homology modeling studies that the catalytic triad of ABHD6 comprises Ser148–His306–Asp278, and the oxyanion hole is formed by Met149 and Phe80. Among the current template structures available, the crystal structure of MenH (2-succinyl-6-hydroxy-2,4-cyclohexadiene-1-carboxylate synthase—a hydrolase from the α/β hydrolase fold family—PDB ID: 2XMZ [26]) resulted in optimal active site geometry for docking studies. The compounds investigated (**1**–**42**), including previously published [19] and new compounds, were modeled using the LigPrep protocol from the Schrödinger Suite software [27]. In order to sample different protonation states of ligands at physiological pH, the Epik module was used [28]. The compounds were docked to the homology model using the standard precision (SP) module Glide from the Schrödinger Suite software. Prior to Glide docking studies, the grid box was centered on the closest active site residues in the case of the ABHD6 model (Phe80, Ser148, Met149, Ile203, His 306). The hydrogen bond constraint “at least one” to main chain amides of oxyanion hole residues Met149 and Phe80 was used. The selected docking poses were used for CoMFA alignment.

### CoMFA studies

The compounds were divided into a training (35 compounds) and a test (7 compounds) set. To construct 3D-QSAR models, both training and test set compounds should span at least four orders of activity magnitude and be well proportioned in each activity magnitude [29]. Both sets covered a reasonable distribution of the biological data. The activity of the compounds was either published elsewhere [19] or is included in the Supplementary Information. The IC_50_ of compounds was not determined experimentally but was calculated as IC_50-single_ from the residual activity according to the following formula as reported previously [30]:

$$ I{C}_{50- single}=\left(I\times R\right)/\left(100-R\right) $$

where *I *denotes the concentration at which residual activity *R* was measured. For compound **42**, with no inhibition, an IC_50_ value of 100,000 nM was assumed. The IC_50_ (nM) values were converted into pIC_50_ values, which were applied as dependent variables for subsequent 3D-QSAR analyses.

Molecular alignment, which has a significant effect on 3D-QSAR models, is the most sensitive factor [29]. In this study, by identifying the binding conformations of the compounds, molecular alignment was obtained through molecular docking. Thus, all the molecules were well aligned in the binding site of ABHD6 for developing the 3D-QSAR model.

The CoMFA model was developed by applying the QSAR module in Sybyl v. 2.1. The standard Tripos force field was used for CoMFA analysis with Gasteiger-Hückel point charges and the default sp^3^ carbon probe with point charge +1.0 [29]. The optimal number of components was designated so that cross-validated *R*^2^ (*Q*^2^) values were maximal and the standard error of prediction was minimal [29].

PLS analysis was applied to correlate the CoMFA fields linearly to pIC_50_ activity values. A cross-validation analysis was performed using the leave-one-out (LOO) method, in which one compound is removed from the data set, and its activity is predicted using the model derived from the remaining compounds [29]. The model resulting in the highest *Q*^2^, optimum number of components (ONC), and the lowest standard error of prediction were taken for further analysis. In addition, the statistical significance of the model was described by the standard error of estimate (SEE) and probability value (F value) [29].

The predictive capability of the 3D-QSAR model was evaluated with the external test set of seven compounds. Moreover, a progressive scrambling validation test was also performed [31, 32]. The test set molecules were also optimized and aligned in the same manner as described above, and their activities were predicted using the model developed.

### Molecular dynamics

MD studies of selected ligand–inhibitor complexes were performed using Desmond v. 3.0.3.1 [33]. The complex was hydrated and ions were added to neutralize protein charges and then to a concentration of 0.15 M NaCl. The complex was minimized and subjected to MD first in the NVT ensemble for 1 ns and then in NPT ensemble for 20 ns with restrictions on the protein backbone in each case. The production run was performed for 100 ns in the NPT ensemble with no restrictions.

## Results and discussion

### The studied compounds

The studied ABHD6 inhibitors **1**–**42** accompanied by ABHD6 inhibitory activity (experimental and predicted) are presented in Table 1. The compounds were divided into training set (35 compounds) and test set (7 compounds: **11**, **17**, **24**, **30**, **34**, **37**, **39**). Most compounds have been published previously [19]; however, some compounds are reported for the first time (**3**, **12**, **27**, **29**–**33**, **38**, **40**, **42**). The synthesis, experimental data and details of the ABHD6 activity determination are presented in the Supplementary Information.

**Table 1:**
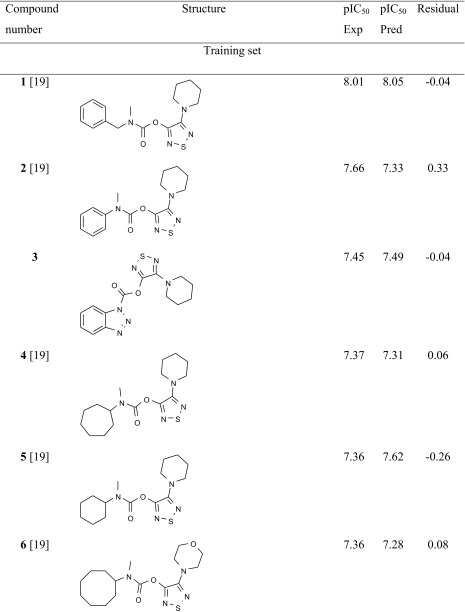

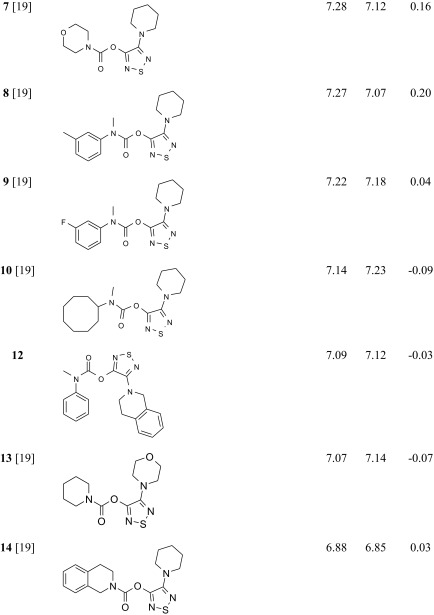

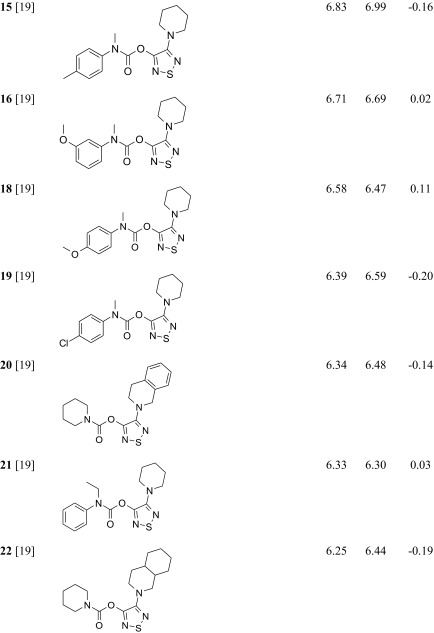

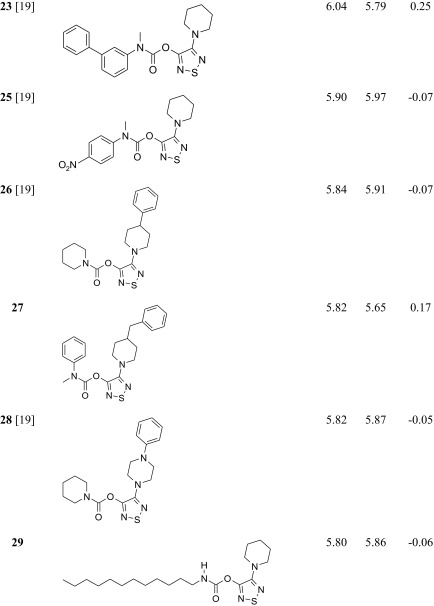

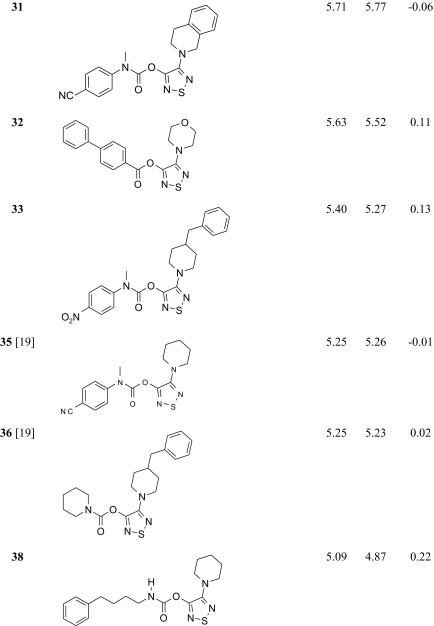

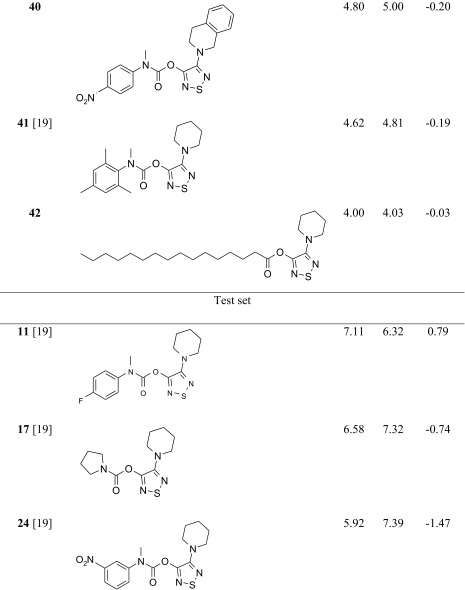

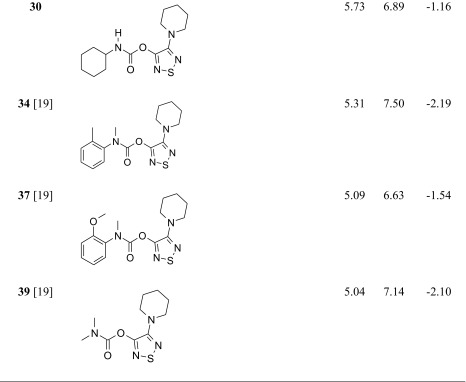
The investigated α/β-hydrolase domain containing 6 (ABHD6) inhibitors with their experimental and predicted pIC_50_ values

### Molecular docking

**Fig. 2:**
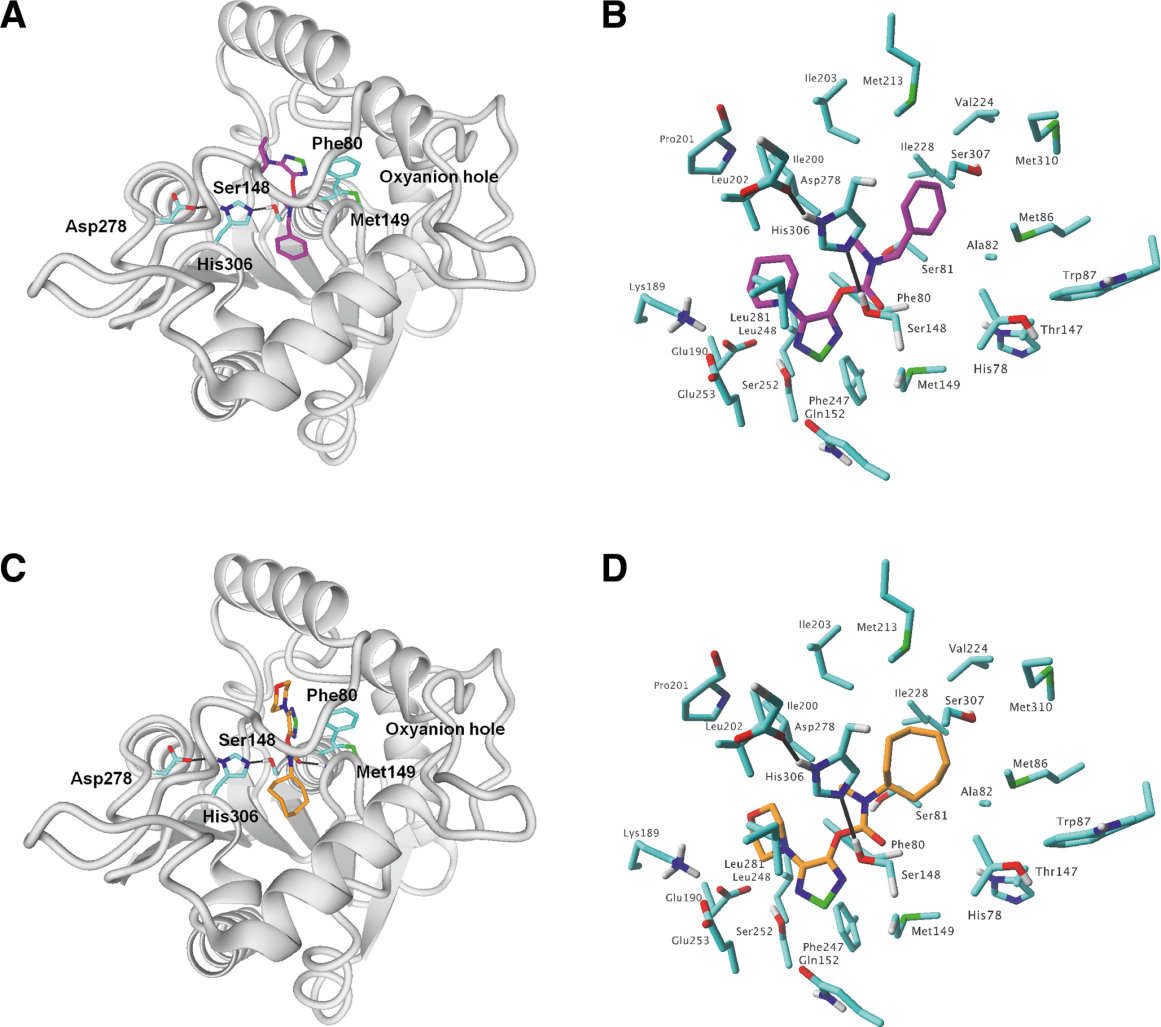
The docking poses of **1** (**a**, **b**) and **6** (**c**, **d**) in the binding pocket of ABHD6 selected for molecular alignment. **a**, **c** Overview of the complex; **b**, **d** details of the binding pocket

Homology models of ABHD6 were previously successfully applied to the molecular docking of inhibitors [19, 25]. Compounds **1**–**42** were thus all docked into the binding site of human ABHD6. Among the Glide docking poses, those in which the carbonyl group interacted with Phe80 were selected and used for molecular alignment. The final binding poses of compounds **1** (the most active compound) and **6** (the most promising compound from our previous article [19]) are presented in Fig. 2. It can be seen that the catalytic triad of ABHD6 comprises Ser148–His306–Asp278, and the oxyanion hole is formed by Met149 and Phe80 [19]. The most important inhibitor contact is a hydrogen bond between the carbonyl group of the ligand and the main chain of Phe80.

### Molecular alignment

The quality of 3D-QSAR models is sensitive to the molecular alignment as inhibitory activity correlates strongly with different substitutions on a specific point in the same compound series [29]. The common structure has been used widely as a base for molecular alignment [20, 24]. However, better results can be obtained when 3D-QSAR models are constructed and evaluated on the active conformations of training and test set compounds, in particular when similar ligands occupy different binding poses in the binding pocket [34]. The molecular alignment of the compounds from the training and test sets is shown in Fig. S1 in the Supplementary Information. The spatial positions of the scaffolds were not kept still in the alignment as is usually the case in docking-based alignment [29]; however, they are within an acceptable range of displacement. This may be caused by the situation that, in reality, different bioactive conformations can be adopted by these derivatives as a result of different substituted groups [29]. Moreover, alignment using docking conformations will also facilitate our understanding of contour maps of the models in a structure-based manner [29].

### CoMFA statistics

**Fig. 3:**
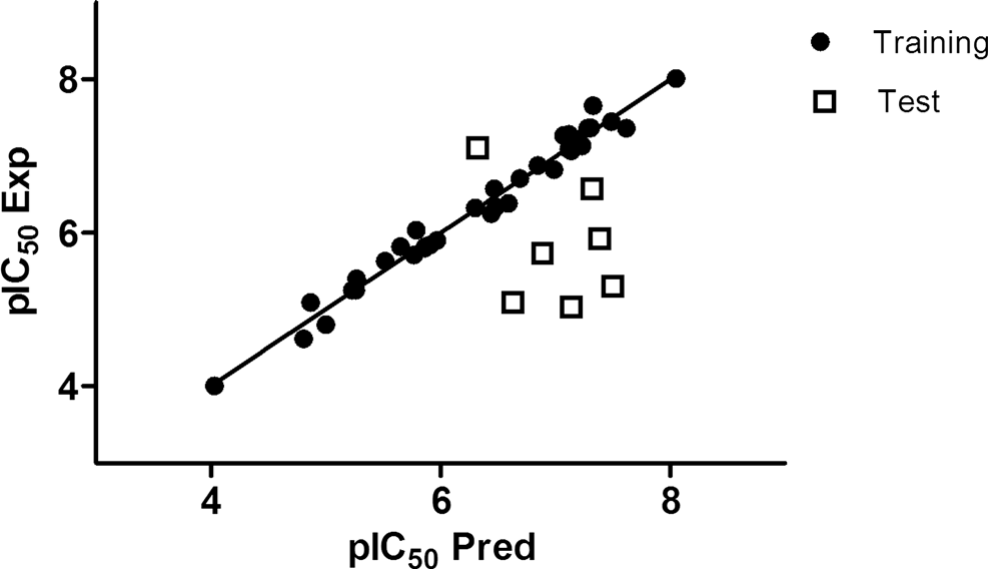
Experimental versus predicted pIC_50_ values for the training and test sets

The 3D-QSAR CoMFA model was built using Sybyl-X v. 2.1. The CoMFA model gave a cross-validated coefficient *Q*^2^ of 0.55 with an optimal component of 4, *R*^2^ of 0.98 and an *F* value of 346.762. The field contributions of parameters were 65.3 % and 34.7 % for the steric field and the electrostatic field descriptor, respectively. These statistical parameters indicate that the CoMFA model is statistically significant. Experimental and predicted IC_50_ values are presented in Table 1. It can be seen that they do not deviate significantly from each other (generally not more than 1 logarithmic unit). Figure 3 shows a very good correlation between the experimental and computed IC_50_ values for the training set, but a worse correlation for the test set. Most compounds from the training set were over-predicted. However, the value of the cross-validated coefficient *Q*^2^ (above 0.5) indicates the good internal predictability of the model.

### Validation of CoMFA model

As the first step in validation, the IC_50_ of the seven compounds from the test set was predicted (Table 1). It can be seen that two most active compounds from the test set (**11** and **17**) are predicted correctly within acceptable error. The activities of the five less active compounds are predicted higher than they should be, probably due to the fact that their IC_50_ was estimated only as IC_50_-single. Furthermore, a progressive scrambling test was performed as an additional validation. The *Q*^2^ statistic returned is an estimate of the predictivity of the model after removing the effects of redundancy [35]. It is computed by fitting the correlation of scrambled to unscrambled data (*R*^2^yy) to the cross-validated correlation coefficient (*Q*^2^) (calculated after each scrambling performed) applying a 3rd order polynomial equation [35]. The cSDEP statistic is an estimated cross-validated standard error at a specific critical point (0.85 default used in this study) for *R*^2^yy, and is computed from a 3rd order polynomial equation that fits the scrambled results [35]. The slope of *Q*^2^ with respect to *R*^2^yy is reported as d*Q*^2^/dR^2^y, and is known as the critical statistic [35]. It shows to what extent the model changes in response to small changes to the dependent variable [35]. In a stable model, d*Q*^2^/d*R*^2^yy should not exceed 1.2 (ideally 1) [35]. This method was employed for the CoMFA model to verify the number of components used to build the model and to check the cross-validation against the possibility of such a redundancy in the training set [35]. Table 2 lists the results of the progressive scrambling of the CoMFA model. *Q*^2^ values above 0.35 are reported to indicate that the original, unperturbed model is robust [32].

**Table 2:** Progressive scrambling test results for the comparative molecular field analysis (CoMFA) model

Component	*Q* ^2^	cSDEP	d*Q* ^2^/d*R* ^2^yy
2	0.49	0.71	−0.16
3	0.49	0.72	0.19
4	0.49	0.74	0.19
5	0.51	0.72	0.21
6	0.48	0.76	0.32
7	0.52	0.74	0.66

### Contour map

**Fig. 4:**
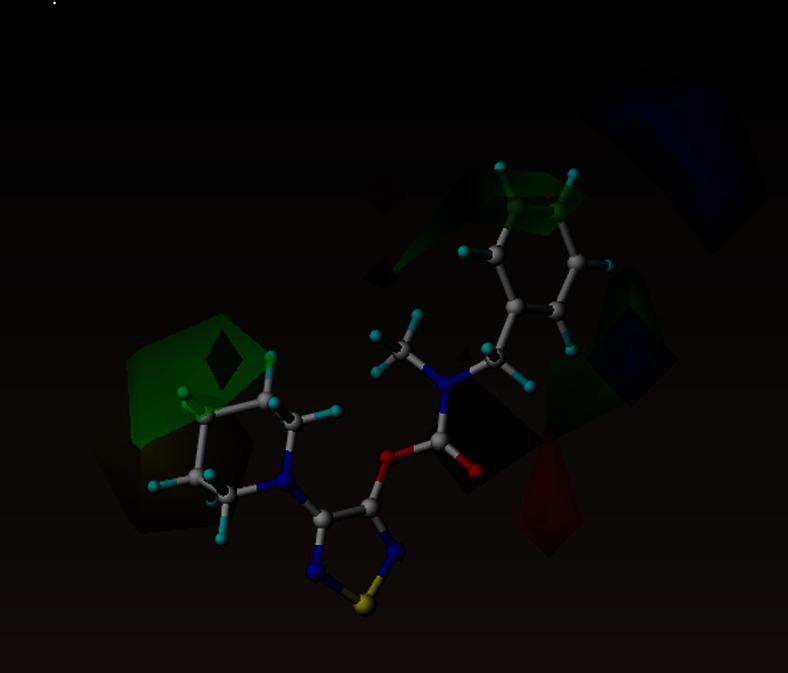
CoMFA steric and electrostatic contour fields. Fields drawn with 85/15 proportion of favorable and unfavorable interactions. The most active compound (**1**) is shown

Figure 4 shows the steric and electrostatic contour maps generated via CoMFA modeling. Steric contour maps give information about the spatial volume of substituted groups at different positions. There were three green and one yellow contour regions located in the active site, with green meaning bulky groups are favored and yellow meaning bulky groups are disfavored. The yellow contour map may explain the lower activity of compounds **27**, **28**, **31**, **33**, **36** and **40,** which have a bulky substituent in this position. Interestingly, there is a red contour region near the carbonyl group, meaning that less negative charge would be favored here. This is probably not connected with the sp^2^ hybridized oxygen atom but with the polarizability of the whole area. Importantly, the carbonyl group is a key moiety interacting with the active site of ABHD6 as the most important inhibitor contact is a hydrogen bond between the carbonyl group of the ligand and the main chain of Phe80 (Fig. 2). This contour may be a false property generated by CoMFA as there is a lack of diversity of compounds in this region, therefore no contours should be present there.

### Directions of inhibitor modifications

On the basis of the constructed CoMFA model, some modifications of compound **1** were suggested. The proposed modifications, i.e., compounds **43**–**47**, are presented in Table 3. It can be concluded that changing the piperidine moiety into morpholine results in the equipotent compound **43**. It was found that introducing an electron-donating group in the meta or preferably the para position of the phenyl group (compounds **44**–**47**) leads generally to enhancement of activity. The strongest effect was observed with an amino group in both meta and para position. In contrast, an electron-withdrawing group resulted in an activity drop (data not presented). A similar effect was found when the N-methyl group was replaced by the N-ethyl or propyl group or when an additional methyl group was added in the benzylic position.

**Table 3:**
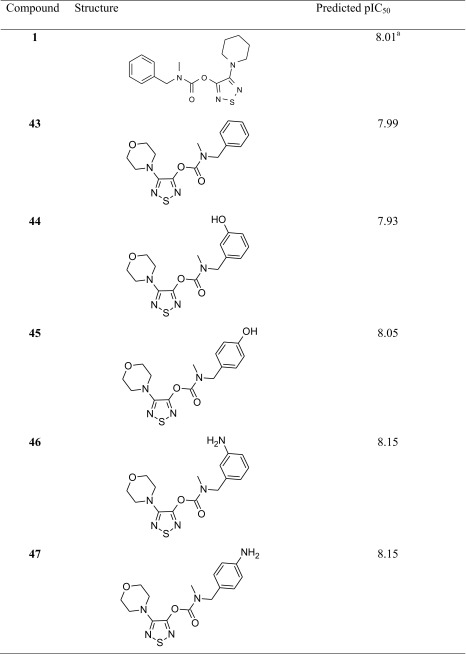
Suggested modifications of compound** 1**. Experimental value^a^

### Molecular dynamics

**Fig. 5:**
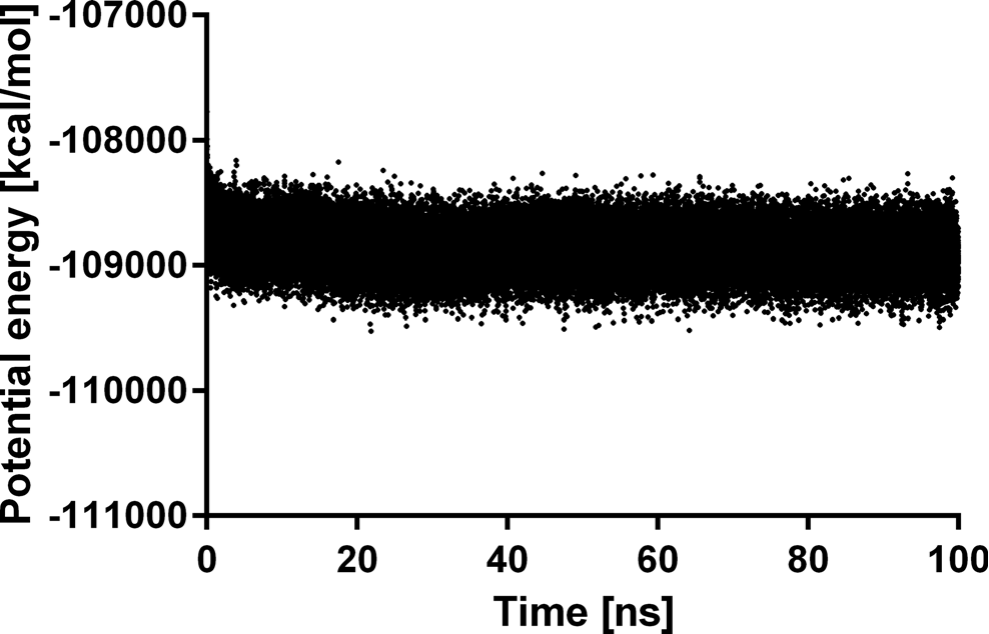
Changes in potential energy of the complex during simulations of the complex of **6** and ABHD6

**Fig. 6:**
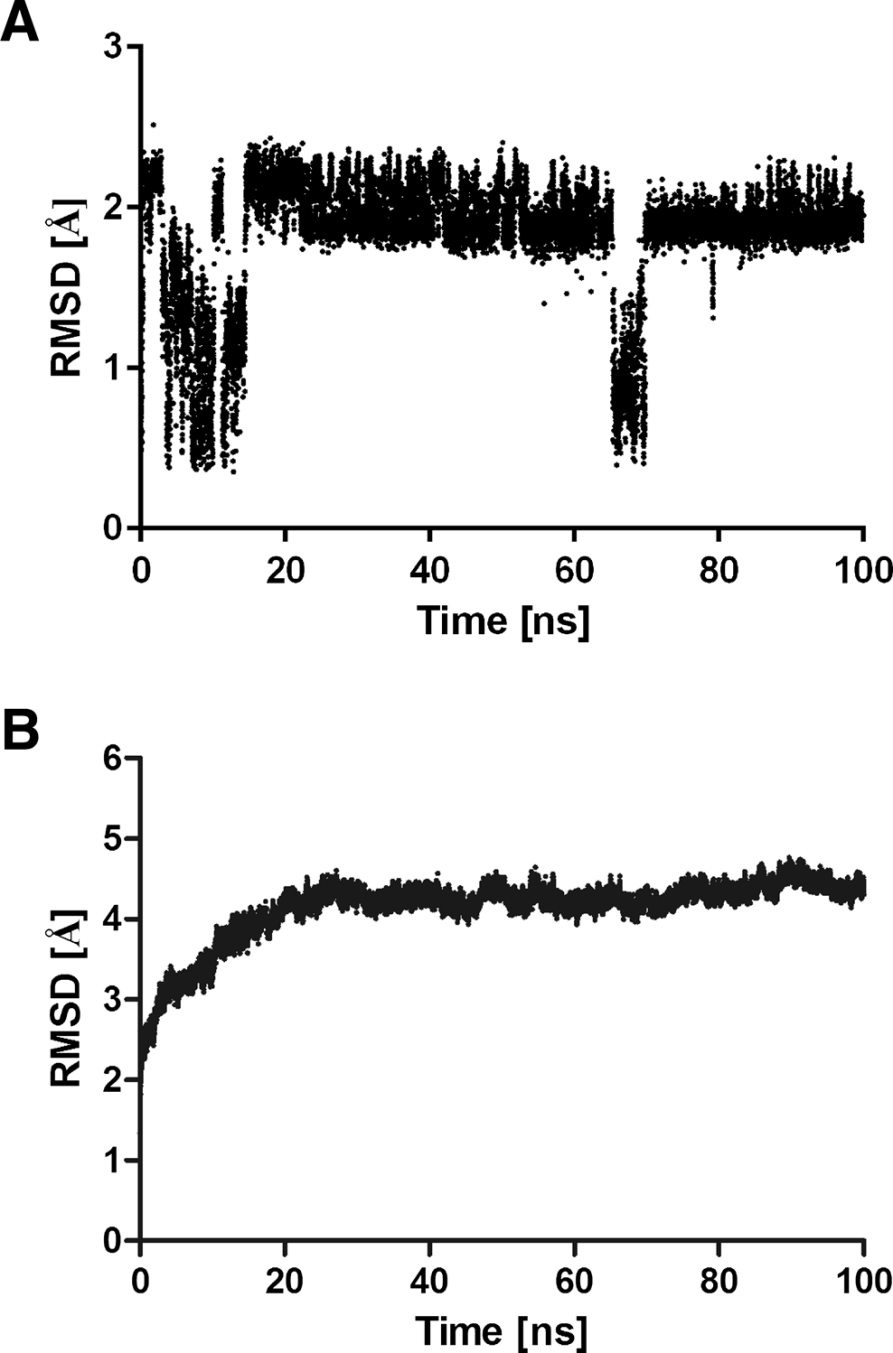
**a**,**b** Root mean square deviations (RMSD) during molecular dynamics (MD) simulations. **a** Inhibitor **6** RMSD compared to initial position. **b** Complex RMSD compared to initial position

**Fig. 7:**
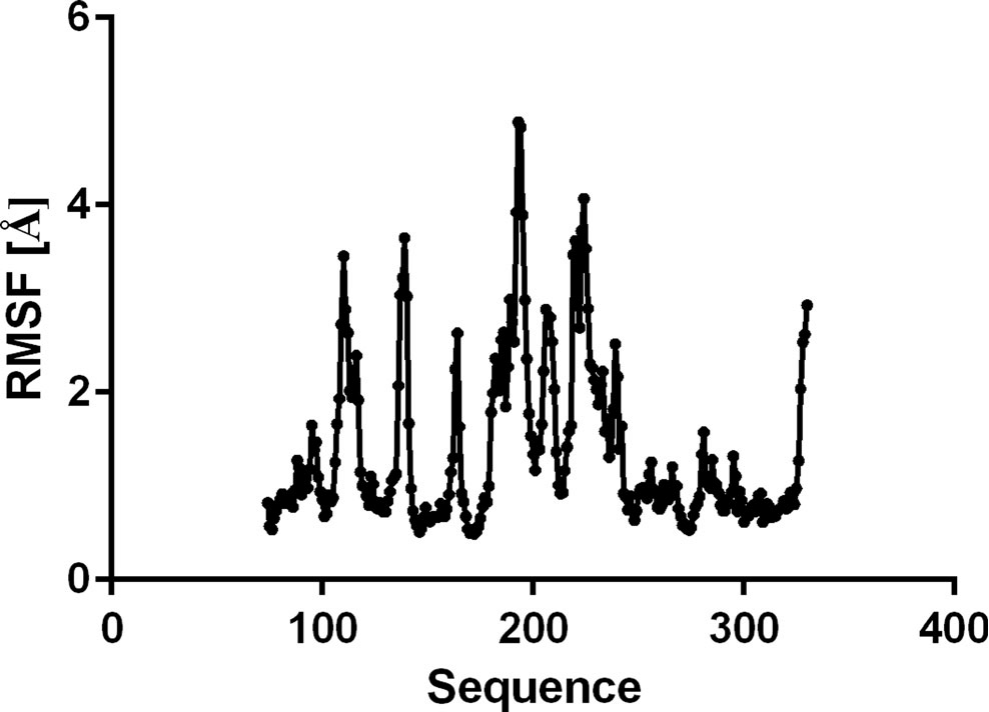
Protein backbone root mean square fluctuation (RMSF) during simulation

**Fig. 8:**
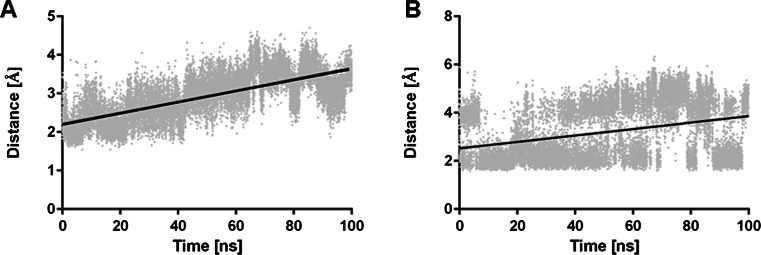
**a**,** b** Distance between the carbonyl group of** 6** and main chain NH of Phe80 (**a**) or side chain of Ser148 (**b**)

The investigated compounds are irreversible inhibitors of ABHD6. In order to study the first events of inhibitor binding, before formation of a covalent bond between the ligand carbonyl group and a side chain of Ser148 [17, 25]. MD simulations were performed for the complex of compound **6** with ABHD6. The complex was stable during simulations, as demonstrated by the decreasing value of potential energy for the complex (Fig. 5) and the complex RMSD (Fig. 6b). The position of **6** in the binding site of ABHD6 was relatively stable, as indicated by the ligand RMSD value below 3 Å (Fig. 6a). Figure 7 shows protein backbone RMSF during simulation. The greatest RMSF value (over 4 Å) has been found for helix end Gly193-Ser194 and Val224. During simulations it was observed that the distance between ligand carbonyl group and the main chain of Phe80 is increasing, weakening the respective hydrogen bond (Fig. 8). In contrast, a bond between the ligand carbonyl group and Ser148 is formed and maintained during the simulation. Although the distance is slightly increasing, there is a possibility to form a covalent bond necessary for irreversible inhibition (Fig. 8).

## Conclusions

We constructed a 3D QSAR model for new inhibitors of ABHD6 that represents the first CoMFA model for this enzyme. The model allowed us to explain the lack of activity of the least active compounds caused by unfavorable steric interactions. Moreover, we performed MD simulations and showed that formation of an additional hydrogen bond between the ligand carbonyl group and Ser148 in the early stages of ligand binding may facilitate the covalent bond formation that is necessary for irreversible inhibition.

## Electronic supplementary material

ESM 1 (DOCX 429 kb)

## Abbreviations

2-AG: 2-Arachidonoylglycerol
ABHD6: α/β Hydrolase domain containing 6
ABHD12: α/β Hydrolase domain containing 12
ANN: Artificial neural network
CB_1_: Cannabinoid receptor 1
CB_2_: Cannabinoid receptor 2
CoMFA: Comparative molecular field analysis
CoMSIA: Comparative molecular similarity index analysis
DSE: Depolarization-induced suppression of excitation
DSI: Depolarization-induced suppression of inhibition
ECS: Endocannabinoid signaling system
G/PLS: Genetic partial least squares
LOO: Leave-one-out
MD: Molecular dynamics
MGL: Monoacylglycerol lipase
ONC: Optimum number of components
PLS: Partial least squares
QSAR: Quantitative structure-activity relationship
SEE: Standard error of estimation
