# Supplementary material for: Comparative molecular field analysis and molecular dynamics studies of α/β hydrolase domain containing 6 (ABHD6) inhibitors
Source: J Mol Model. 2015 Sep 8;21(10):250. doi: 10.1007/s00894-015-2789-8 (PMC4562993; doi:10.1007/s00894-015-2789-8)
Supplement: Supplementary file 1 — (DOCX 429 kb) [file 894_2015_2789_MOESM1_ESM.docx]

**Comparative Molecular Field Analysis (CoMFA) and molecular dynamics studies of α/β hydrolase domain containing 6 (ABHD6) inhibitors**

Agnieszka A. Kaczor^1,2^ ^*^, Katarzyna M. Targowska-Duda^3^, Jayendra Z. Patel^2^, Tuomo Laitinen^2^, Teija Parkkari^2^, Yahaya Adams^2^, Tapio J. Nevalainen^2^, Antti Poso^2^

*^1^Department of Synthesis and Chemical Technology of Pharmaceutical Substances with Molecular Modeling Lab, Faculty of Pharmacy with Division of Medical Analytics, Medical University of Lublin, 4a Chodźki St., PL-20059, Lublin, Poland*

*^2^School of Pharmacy, University of Eastern Finland, Yliopistonranta 1C, P.O. Box 1627, FI-70211 Kuopio, Finland*

*^3^Department of Biopharmacy, Faculty of Pharmacy with Division of Medical Analytics, Medical University of Lublin, 4a Chodźki St., PL-20059, Lublin, Poland*

## Supplementary material

### Contents

| **Fig. S1.** Alignment of 42 compounds in the training and test sets based on the molecular docking results to ABHD6 binding site. Non-polar hydrogen atoms omitted for clarity. | S2 |
| --- | --- |
| General scheme and procedure for the preparation of thiadiazole carbamates/esters (**3, 12, 27, 29-33, 38, 40** and **42**) | S3-S4 |
| Spectroscopic characterisation of compounds **3, 12, 27, 29-33, 38, 40** and **42** | S4-S9 |
| Determination of ABHD6 activity using a sensitive fluorescent  glycerol assay for the compounds **3, 12, 27, 29-33, 38, 40** and **42** | S9 |
| Inhibitory activities of novel 1,2,5-thiadiazole carbamates/esters **3, 12, 27, 29-33, 38, 40** and **42** against ABHD6 (Table S1) | S10 |
| References | S10-S11 |


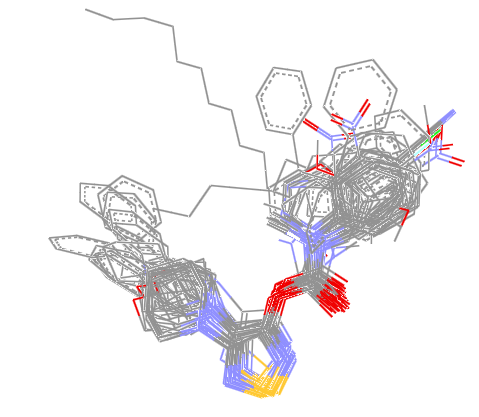


**Fig. S1.** Alignment of 42 compounds in the training and test sets based on the molecular docking results to ABHD6 binding site. Non-polar hydrogen atoms omitted for clarity.

**General scheme and procedure for the preparation of thiadiazole carbamates/esters** (**3, 12, 27, 29-33, 38, 40** **and 42**):

**Scheme 1.** Synthesis of 1,2,5-thiadiazole derivatives **3, 12, 27, 29-33, 38, 40** and **42***^a^*

*^

^*

*^a^* Reagents and conditions: (a) dry THF, KOtBu, 0-25 ^o^C, 16-24 h; (b) dry THF, Reflux, 2-6 h; (c) see (a).

**General procedures for preparation of 1,2,5-thiadiazole carbamates (3, 12, 27, 31, 33 and 40)**

As per our earlier reported procedure [1] 3,4-dichloro-1,2,5-thiadiazole was coupled with the appropriate secondary amine to afford a corresponding monochloro 1,2,5-thiadiazole derivative, which was then converted to 1,2,5-thiadiazole alcohol (I) via treatment with aqueous alkali. Finally, coupling with appropriate carbamoyl chloride (II), gave the desired 1,2,5-thiadiazole carbamates (**3, 12, 27, 31, 33** and **40**).

**General procedures for preparation of 1,2,5-thiadiazole carbamates (29, 30 and 38)**

A mixture of appropriate 1,2,5-thiadiazole alcohol (1.0 equiv, I) and isocyanate (1.0 equiv, III) in dry THF (0.2 M) was refluxed for 2-6 h. The progress of the reaction was monitored by TLC using 20% EtOAc in PE as a mobile phase. Reaction mixture was diluted with EtOAc. It was washed with H_2_O and brine. The organic layer was dried over sodium sulphate, filtered and concentrated under vacuum to afford crude 1,2,5-thiadiazole carbamates which were purified by flash column chromatography using PE : EtOAc (9: 1) as an eluent. The desired fractions were collected and solvents were evaporated on a rotatory evaporator to afford pure 1,2,5-thiadiazole carbamates **(29, 30** and **38)**.

**General procedures for preparation of 1,2,5-thiadiazole esters (32 and 42)**

1,2,5-thiadiazole alcohol (1.0 equiv, I) was coupled with acid chloride (1.15 equiv., IV) in a similar manner as described for the preparation of compounds **3, 12, 27, 31, 33** and **40** to afford 1,2,5-thiadiazole esters (**32** and **42**).

**Spectroscopic characterisation of 1,2,5-thiadiazole carbamates/esters (3, 12, 27, 29-33, 38, 40 and 42)**

**4-(Piperidin-1-yl)-1,2,5-thiadiazol-3-yl 1*H*-benzo[*d*][1,2,3]triazole-1-carboxylate (3)**

**

**

Light-yellow solid (70 mg, 8%); ^1^H NMR (CDCl_3_): *δ* 8.09 (d, *J* = 8.35 Hz, 1H), 7.97 (d, *J* = 8.35 Hz, 1H), 7.59 (t, *J* = 7.30 Hz, 1H), 7.44 (t, *J* = 7.65 Hz, 1H), 3.84-3.78 (br s, 4H), 1.79-1.71 (br s, 6H); ^13^C NMR (CDCl_3_): *δ* 149.46, 145.37, 133.23, 129.18, 125.07 (2C), 119.80 (2C), 113.45, 48.48 (2C), 25.52, 24.35, 24.31; Anal. Calcd for C_14_H_14_N_6_O_2_S: C, 50.90; H, 4.27; N, 25.44%. Found: C, 50.92; H, 4.31; N, 25.47%; ESI-MS: 353.12 [M + Na]^+^

**4-(3,4-Dihydroisoquinolin-2(1*H*)-yl)-1,2,5-thiadiazol-3-yl-(4-phenylbutyl)carbamate**

**(12)**

**

**

White solid (323 mg, 69%); ^1^H NMR (CDCl_3_): *δ* 7.48-7.38 (m, 5H), 7.18-7.13 (m, 3H), 7.01-6.98 (m, 1H), 4.52-4.47 (br s, 2H), 3.58-3.54 (br s, 2H), 3.46-4.41 (br s, 3H), 2.86-2.82 (br s, 2H); ^13^C NMR (CDCl_3_): *δ* 152.37, 150.92, 145.66, 141.99, 133.93, 133.24, 129.43, 128.67 (2C), 127.72, 126.43 (2C), 126.26 (2C), 126.09, 49.55, 45.18, 38.71, 28.45; Anal. Calcd for C_19_H_18_N_4_O_2_S: C, 62.28; H, 4.95; N, 15.29%. Found: C, 62.31; H, 4.93; N, 15.27%; ESI-MS: 367.04 [M + H]^+^

**4-(4-Benzylpiperidin-1-yl)-1,2,5-thiadiazol-3-yl methyl(phenyl)carbamate (27)**

**

**

White solid (270 mg, 61%); ^1^H NMR (CDCl_3_): *δ* 7.38-7.14 (m, 10H), 3.74-3.68 (br s, 2H), 3.42-3.37 (br s, 3H), 2.67-2-63 (br s, 2H), 2.55-2.51 (br s, 2H), 1.60-1.56 (m, 3H), 1.27-1.23 (m, 2H); ^13^C NMR (CDCl_3_): *δ* 153.14, 150.92, 146.08, 141.96, 140.07, 129.29, 129.02 (3C), 128.83, 128.18 (3C), 127.96, 126.39, 48.15 (2C), 43.02, 37.67, 31.52 (3C); Anal. Calcd for C_22_H_24_N_4_O_2_S: C, 64.68; H, 5.92; N, 13.71%. Found: C, 64.67; H, 5.91; N, 13.68%; ESI-MS: 409.13 [M + H]^+^

**4-(Piperidin-1-yl)-1,2,5-thiadiazol-3-yl (4-phenylbutyl)carbamate (29)**

**

**

White solid (172 mg, 80%); ^1^H NMR (CDCl_3_): *δ* 8.55-8.51 (br s, -NH), 3.71-3.64 (br s, 4H), 3.39-3.35 (q, *J* = 6.63 Hz, 2H), 1.66-1.61 (br s, 6H), 1.55-1.60 (m, 2H), 1.38-1.17 (m, 18H), 0.86 (t, *J* = 6.95 Hz, 3H); ^13^C NMR (CDCl_3_): *δ* 156.30, 149.31, 148.80, 46.95 (2C), 40.28, 31.89, 29.59, 29.54, 29.44, 29.36, 29.31, 29.17, 26.77, 25.48 (2C), 22.66, 14.09; Anal. Calcd for C_20_H_36_N_4_O_2_S: C, 60.57; H, 9.15; N, 14.13%. Found: C, 60.54; H, 9.13; N, 14.15%; ESI-MS: 396.66 [M + H]^+^

**4-(Piperidin-1-yl)-1,2,5-thiadiazol-3-yl cyclohexylcarbamate (30)**

**
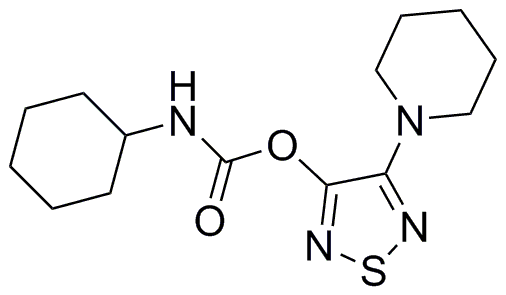
**

White solid (140 mg, 84%); ^1^H NMR (DMSO): *δ* 8.47 (d, *J* = 7.6 Hz, -NH), 3.68-3.63 (m, 5H), 1.81-1.78 (m, 2H), 1.69-1.61 (m, 2H), 1.62-1.49 (m, 7H), 1.44-1.34 (m, 4H), 1.26-1.22 (m, 1H); ^13^C NMR (CDCl_3_): *δ* 156.30, 148.88, 148.43, 49.21, 46.95 (2C), 32.68 (2C), 25.50 (3C), 25.38, 24.39, 24.31; Anal. Calcd for C_14_H_22_N_4_O_2_S: C, 54.17; H, 7.14; N, 18.05%. Found: C, 54.18; H, 7.11; N, 18.07%; ESI-MS: 310.81 [M + H]^+^

**4-(3,4-Dihydroisoquinolin-2(1*H*)-yl)-1,2,5-thiadiazol-3-yl-(4-cyanophenyl)(methyl)carbamate (31)**

**

**

Off-white solid (448 mg, 82%); ^1^H NMR (CDCl_3_): 7.71 (d, *J* = 8.3 Hz, 2H), 7.51 (d, *J* = 8.3 Hz, 2H), 7.19-7.12 (m, 3H), 7.01-6.98 (m, 1H), 4.55-4.51 (br s, 2H), 3.65-3.61 (m, 2H), 3.52-3.48 (br s, 3H), 2.90-2.86 (br s, 2H); ^13^C NMR (CDCl_3_): *δ* 152.63, 150.63, 145.94, 145.31, 133.84, 133.29 (2C), 133.11, 128.84 (2C), 126.72 (2C), 126.36 (2C), 126.27, 118.03, 49.78, 45.51, 38.16, 29.52; Anal. Calcd for C_20_H_17_N_5_O_2_S: C, 61.37; H, 4.38; N, 17.89%. Found: C, 61.35; H, 4.39; N, 17.91%; ESI-MS: 392.04 [M + H]^+^

**4-Morpholino-1,2,5-thiadiazol-3-yl [1,1'-biphenyl]-4-carboxylate (32)**

**

**

White solid (231 mg, 89%); ^1^H NMR (CDCl_3_): *δ* 8.22 (d, *J* = 8.2 Hz, 2H), 7.76 (d, *J* = 8.2 Hz, 2H), 7.65 (d, *J* = 7.5 Hz, 2H), 7.51-7.42 (m, 3H), 3.76 (t, *J* = 4.55 Hz, 4H), 3.48 (t, *J* = 4.55 Hz, 4H); ^13^C NMR (CDCl_3_): *δ* 162.98, 153.28, 147.49, 145.97, 139.44, 131.01 (2C), 129.10 (2C), 128.67, 127.65 (2C), 127.36 (2C), 126.34, 66.33 (2C), 48.14 (2C); Anal. Calcd for C_19_H_17_N_3_O_3_S: C, 62.11; H, 4.66; N, 11.44%. Found: C, 62.09; H, 4.65; N, 11.45 %; ESI-MS: 368 [M + H]^+^

**4-(4-Benzylpiperidin-1-yl)-1,2,5-thiadiazol-3-yl methyl(4-nitrophenyl)carbamate**

**(33)**

**

**

Brown viscous oil (132 mg, 38%); ^1^H NMR (CDCl_3_): *δ* 8.26 (d, *J* = 8.4 Hz, 2H), 7.55 (d, *J* = 8.1 Hz, 2H), 7.30-7.12 (m, 5H), 3.82-3.78 (br s, 2H), 3.52-3.48 (br s, 3H), 2.80-2.75 (m, 2H), 2.55-2.51 (m, 2H), 1.69-1.59 (m, 4H), 1.28-1.21 (m, 1H); ^13^C NMR (CDCl_3_): *δ* 153.32, 150.59, 147.61, 145.61, 139.98, 129.09 (3C), 128.33 (3C), 126.08, 125.87, 124.70 (2C), 48.42 (2C), 43.03, 38.14, 31.52 (3C); Anal. Calcd for C_22_H_23_N_5_O_4_S: C, 58.27; H, 5.11; N, 15.44%. Found: C, 58.30; H, 5.12; N, 15.47%; ESI-MS: 454.10 [M + H]^+^

**4-(Piperidin-1-yl)-1,2,5-thiadiazol-3-yl (4-phenylbutyl)carbamate (38)**

**

**

Light yellow solid (150 mg, 77%); ^1^H NMR (CDCl_3_): *δ* 8.55-8.51 (br s, -NH), 7.27-7.25 (m, 2H), 7.17-7.14 (m, 3H), 3.68-3.63 (br s, 4H), 3.42-3.38 (q, *J* = 6.5 Hz, 2H), 2.63 (t, *J* = 7.35 Hz, 2H), 1.70-1.61 (m, 10H); ^13^C NMR (CDCl_3_): *δ* 156.30, 149.35, 148.76, 141.83, 128.38 (2C), 128.34 (2C), 125.85, 46.95 (2C), 40.08, 35.38, 28.97, 28.46, 25.48 (2C), 24.31; Anal. Calcd for C_18_H_24_N_4_O_2_S: C, 59.98; H, 6.71; N, 15.54%. Found: C, 60.02; H, 6.72; N, 15.53%; ESI-MS: 360.77 [M + H]^+^

**4-(3,4-Dihydroisoquinolin-2(1*H*)-yl)-1,2,5-thiadiazol-3-yl-(4-phenylbutyl)carbamate**

**(40)**

**

**

Yellow viscous oil (512 mg, 90%); ^1^H NMR (CDCl_3_): *δ* 8.27 (d, *J* = 8.7 Hz, 2H), 7.58 (d, *J* = 8.6 Hz, 2H), 7.18-7.12 (m, 3H), 7.01-6.97 (m, 1H), 4.57-4.53 (br s, 2H), 3.67-3.63 (m, 2H), 3.57-3.51 (br s, 3H), 2.93-2.88 (br s, 2H); ^13^C NMR (CDCl_3_): *δ* 152.66, 150.64, 147.58, 145.26, 133.83, 133.08, 128.84 (2C), 126.73 (2C), 126.36, 126.26, 125.90, 124.76 (2C), 49.79, 45.54, 38.21, 28.54; Anal. Calcd for C_19_H_17_N_5_O_4_S: C, 55.47; H, 4.16; N, 17.02%. Found: C, 55.45; H, 4.18; N, 17.01%; ESI-MS: 412.02 [M + H]^+^

**4-(Piperidin-1-yl)-1,2,5-thiadiazol-3-yl palmitate (42)**

**

**

Light yellow solid (360 mg, 79%); ^1^H NMR (CDCl_3_): *δ* 3.38-3.33 (br s, 4H), 2.57 (t, *J* = 7.45 Hz, 2H), 1.75-1.70 (m, 2H), 1.63-1.61 (m, 6H), 1.39-1.24 (m, 24H), 0.86 (t, *J* = 6.75 Hz, 3H); ^13^C NMR (CDCl_3_): *δ* 170.01, 153.81, 146.08, 49.01 (2C), 34.14, 31.90, 29.66 (3C), 29.64, 29.61, 29.55, 29.38, 29.34, 29.17, 28.98, 25.38 (2C), 24.54, 24.16, 22.67, 14.09; Anal. Calcd for C_23_H_41_N_3_O_2_S: C, 65.21; H, 9.75; N, 9.92;%. Found: C, 65.19; H, 9.78; N, 9.89;%; ESI-MS: 462.74 [M + K]^+^

**Determination of ABHD6 activity using a sensitive fluorescent glycerol assay for the compounds 3, 12, 27, 29-33, 38, 40 and 42**

Glycerol production from 1-AG hydrolysis was determined with a previously validated sensitive fluorescent glycerol assay [2,3]. Briefly, glycerol production was coupled via a three-step enzymatic cascade to hydrogen peroxide (H_2_O_2_) dependent generation of resorufin whose fluorescence (λ_ex_ 530; λ_em_ 590 nm) was kinetically monitored using a Tecan Infinite M200 plate reader (Tecan Group Ltd., Männedorf, Switzerland). In short, lysates of HEK293 cells with transient overexpression of hABHD6 (99 µL, 0.3 µg protein/well) were pretreated for 30 min with the solvent (DMSO) or the indicated concentration of the inhibitor (1 µL), after which 1-AG (100 µL, 12.5 µM final concentration) was added and the reaction kinetically monitored for 90 min. The assays routinely contained 0.5% (w/v) BSA (essentially fatty acid free) as a carrier for lipophilic compounds. 1-AG was used instead of 2-AG, as this is the preferred endocannabinoid isomer for hABHD6 and hABHD12 [3]. The IC_50_-values at time-point 90 min were calculated after nonlinear fitting of the inhibitor dose-response curves. Assay blanks without enzyme were included in each experiment and fluorescence of the assay blank was subtracted before calculation of the final results.

**Table S1. Inhibitory activities of novel 1,2,5-thiadiazole carbamates/esters 3, 12, 27, 29-33, 38, 40 and 42 against ABHD6**

| Compound | hABHD6  (pI_50_ ± SEM [IC_50,_ μm]) *^a^* or (% inhibition at 1 μm) *^b^* | Compound | hABHD6  (pI_50_ ± SEM [IC_50,_ μm]) *^a^* or (% inhibition at 1 μm) *^b^* |
| --- | --- | --- | --- |
| **3** | 7.45 ± 0.04 [0.035] | **33** | 20 % |
| **12** | 7.01 ± 0.07 [0.098] | **38** | 11 % |
| **27** | 40 % | **40** | 6 % |
| **29** | 39 % | **42** | NI *^c^* |
| **30** | 35 % | WWL70 *^e^* | 7.07 ± 0.05 [0.085] |
| **31** | 34 % | THL *^f^* | 7.32 ± 0.11 [0.048] |
| **32** | 30 % | JZP-430 (**6**) *^g^* | 7.36 ± 0.05 [0.044] |

*^a^* pI_50_ values (-log_10_ [IC_50_]) represent the mean ± S.E.M. from three independent experiments performed in duplicates. IC_50_ values are calculated for those compounds having ≥50% inhibition at 1 μM for hABHD6; and are derived from the mean pI_50_ values as shown in brackets. *^b^* The percentage (%) of inhibition is represented as the mean from two independent experiments performed in duplicates. *^c^* NI indicates no inhibition. *^d^* WWL70, *N*-Methyl-*N*-[[3-(4-pyridinyl)phenyl]methyl]-4'-(aminocarbonyl)[1,1'-biphenyl]-4-yl carbamic acid ester is a reference inhibitor and inhibitory value is as per our earlier observation reported in ref. [2]. *^e^* THL, tetrahydrolipstatin (orlistat) reported in ref. [2]. *^f^* Reported in ref. [1].

References

1. Patel JZ, Nevalainen TJ, Savinainen JR, Adams Y, Laitinen T, Runyon RS, Vaara M, Ahenkorah S, Kaczor AA, Navia-Paldanius D, Gynther M, Aaltonen N, Joharapurkar AA, Jain MR, Haka AS, Maxfield FR, Laitinen JT, Parkkari T (2015) Optimization of 1,2,5-thiadiazole carbamates as potent and selective ABHD6 inhibitors. ChemMedChem 10:253–265.

2. Aaltonen N, Savinainen JR, Ribas CR, Rönkkö J, Kuusisto A, Korhonen J, Navia-Paldanius D, Häyrinen J, Takabe P, Käsnänen H, Pantsar T, Laitinen T, Lehtonen M, Pasonen-Seppänen S, Poso A, Nevalainen T, Laitinen JT (2013) Piperazine and piperidine triazole ureas as ultrapotent and highly selective inhibitors of monoacylglycerol lipase. Chem Biol 20:379–390

3. Navia-Paldanius D, Savinainen JR, Laitinen JT (2012) Biochemical and pharmacological characterization of human α/β-hydrolase domain containing 6 (ABHD6) and 12 (ABHD12). J Lipid Res 53:2413–2424
